# A sterile hydroponic system for characterising root exudates from specific root types and whole-root systems of large crop plants

**DOI:** 10.1186/s13007-018-0380-x

**Published:** 2018-12-20

**Authors:** Akitomo Kawasaki, Shoko Okada, Chunyan Zhang, Emmanuel Delhaize, Ulrike Mathesius, Alan E. Richardson, Michelle Watt, Matthew Gilliham, Peter R. Ryan

**Affiliations:** 1 CSIRO Agriculture and Food, Canberra, ACT Australia; 2 CSIRO Land and Water, Canberra, ACT Australia; 3 Division of Plant Science, Research School of Biology, Australian National University, Canberra, ACT Australia; 4 Forschungszentrum Jülich GmbH, Jülich, Germany; 5 ARC Centre of Excellence in Plant Energy Biology, School of Agriculture, Food and Wine, Waite Research Institute, University of Adelaide, Glen Osmond, SA Australia; 6 Present Address: College of Life Science, China West Normal University, Nanchong, Sichuan China

**Keywords:** Hydroponic, Root exudates, Organic anion, Malate, Aluminium-tolerance, Wheat, Barley, Sterile system, *TaALMT1*

## Abstract

**Background:**

Plant roots release a variety of organic compounds into the soil which alter the physical, chemical and biological properties of the rhizosphere. Root exudates are technically challenging to measure in soil because roots are difficult to access and exudates can be bound by minerals or consumed by microorganisms. Exudates are easier to measure with hydroponically-grown plants but, even here, simple compounds such as sugars and organic acids can be rapidly assimilated by microorganisms. Sterile hydroponic systems avoid this shortcoming but it is very difficult to maintain sterility for long periods especially for larger crop species. As a consequence, studies often use small model species such as *Arabidopsis* to measure exudates or use seedlings of crop plants which only have immature roots systems.

**Results:**

We developed a simple hydroponic system for cultivating large crop plants in sterile conditions for more than 30 days. Using this system wheat (*Triticum aestivum* L.) and barley (*Hordeum vulgare* L.) plants were grown in sterile conditions for 30 days by which time they had reached the six-leaf stage and developed mature root systems with seminal, nodal and lateral roots. To demonstrate the utility of this system we characterized the aluminium-activated exudation of malate from the major types of wheat roots for the first time. We found that all root types measured released malate but the amounts were two-fold greater from the seminal and nodal axile roots compared with the lateral roots. Additionally, we showed that this sterile growth system could be used to collect exudates from intact whole root systems of barley.

**Conclusions:**

We developed a simple hydroponic system that enables cereal plants to be grown in sterile conditions for longer periods than previously recorded. Using this system we measured, for the first time, the aluminium-activated efflux of malate from the major types of wheat roots. We showed the system can also be used for collecting exudates from intact root systems of 30-day-old barley plants. This hydroponic system can be modified for various purposes. Importantly it enables the study of exudates from crop species with mature root systems.

**Electronic supplementary material:**

The online version of this article (10.1186/s13007-018-0380-x) contains supplementary material, which is available to authorized users.

## Background

Cereal plants are estimated to release approximately 11% of their net fixed carbon from the roots as rhizodeposits [1]. These deposits include sloughed off border cells and tissues, mucilage, volatile organic molecules and exudates comprised of high and low molecular weight compounds such as sugars, amino acids, phenolics, and organic anions [1–3]. Collectively these compounds modify the rhizosphere and affect the composition and abundance of microorganisms [4, 5]. Plants are also known to release specific exudate compounds in response to certain environmental stresses. A well-studied example is the release of organic anions from root apices in response to the toxic aluminium (Al^3+^) ions prevalent in acid soils. These anions protect the sensitive root apices by chelating the toxic Al^3+^ cations in the apoplast of root cells to form less-harmful complexes [6]. In wheat this response is controlled by two genes: *TaALMT1* on chromosome 4DL and *TaMATE1B* on chromosome 4BL. *TaALMT1* encodes an anion channel from the aluminium-activated malate transporter family that releases malate anions [7] while *TaMATE1B* encodes a transporter from the multidrug and toxic compound exudation (MATE) family that releases citrate anions [8, 9]. Both TaALMT1 and TaMATE1B are constitutively expressed in the root apices of wheat. However, TaALMT1 requires soluble Al^3+^ cations to activate malate release at low pH, whereas citrate release via TaALMT1B is constitutive and independent of external pH or Al^3+^ concentration.

Various methods have been used to collect root exudates and they all have advantages and disadvantages. Exudates can be collected directly from the soil adjacent to roots using suction cups with polymer or ceramic filters [10–12] but it is unclear how the samples collected this way have been modified by microorganisms and, therefore, how well they reflect the compounds released from the roots. Hydroponic systems are convenient for measuring root exudates because they avoid the mechanical damage incurred by removing roots from solid substrates and minimise the microbial degradation of exudates that will occur in soil [13]. Non-sterile hydroponic systems have been successfully employed to measure certain root exudates [14–17], but this becomes more difficult in mature plants because contamination is inevitable. Therefore, the composition and quantity of exudates collected in non-sterile hydroponics growth systems will be affected by microbial activity as well [18].

Sterile hydroponic systems enable root exudates to be measured under controlled conditions. These systems are generally suitable for seedlings or small plants like *Arabidopsis* that can be grown easily in low-volume wells [19] or sterile glass flasks [20]. It is technically difficult to maintain sterility much beyond the seedling stage with larger plants including many crop species [13, 21]. The reason for this is that the whole plant needs to be fully enclosed to prevent contamination and the roots require larger volumes of hydroponic solution to match their nutrient needs. The solutions need to be renewed periodically and constantly aerated to avoid anaerobic conditions. Both of these requirements increase the risk of contamination. Consequently, most previous studies that have examined the exudates from crop species (e.g. wheat, barley and maize) in sterile conditions have used young plants with immature root systems [22, 23]. The well-characterised organic anion release from Al-resistant wheat plants has only been performed on the seminal roots of young seedlings [7, 9, 24–27] and it is unknown whether the same response occurs in the nodal or lateral roots of older plants. This is an important omission since the composition and quantity of exudates can change with plant age [28] and seminal roots represent a small fraction of the total root system of a mature plant.

A few studies claim to have maintained sterile root systems on wheat and maize plants for several weeks. For instance, Gaume et al. [29, 30] studied root exudates from 18- and 21-day-old maize plants, and Warren [31, 32] examined root exudates from 28-day-old wheat plants. However, it is very unlikely that either of these studies maintained truly sterile root systems over those periods because the shoots were not fully enclosed in sterile chambers. There is a need to develop new methods for analysing exudates from the various root types on mature crop plants under sterile conditions.

We describe a simple and novel hydroponic system that is fully-enclosed and able to maintain crop plants like wheat and barley in sterile conditions until at least the six-leaf stage. Nodal roots and an extensive lateral root system were able to develop during this time. To demonstrate the utility of this method, we characterised the Al^3+^-activated malate exudation from the seminal, nodal and lateral roots for the first time. We show that this system is also suitable for collecting exudates from the whole intact root systems from similarly-aged sterile plants.

## Results

### A system for growing large crop plants under sterile conditions for 30 days

A hydroponic system was developed for growing cereals such as wheat and barley under axenic conditions for at least 30 days, which was sufficient for plants to develop mature root systems comprising seminal, nodal and lateral roots.

*Surface sterilising the seed* An important obstacle in maintaining sterility was ensuring the seed was effectively surface-sterilized prior to germination. Using wheat grain from different batches or sources several methods were tested which included various combinations of ethanol, bleach and H_2_O_2_ washes, fumigation with chlorine gas and heat treatments. Sterility was easier to achieve if the grain were freshly harvested, clean and visibly free from fungal contamination. Bacteria were relatively easy to control and a single treatment with bleach or chlorine gas was sufficient to eliminate bacterial growth. The fungal spores were more resilient and could not always be controlled with a single treatment with bleach or chlorine gas. Fungal contamination was sometimes detected many days after germination. Endophytes were difficult to eliminate because they were embedded in tissues, but as long as they did not emerge from the rhizodermis and infect the solution they did not affect exudate composition. The most effective procedure for preventing fungal contamination started by imbibing the seed and then using a series of bleach and H_2_O_2_ washes followed by a 50 °C heat treatment (see “Methods” section). This procedure reduced germination rates by 5–28%, depending on the seed batch, however the sterilized seedlings developed 130–213% longer seminal roots (*P* < 0.05) than the non-sterilized seeds after 14 days on agar/nutrient plates and the plants later grew well. The method was similarly effective in sterilising barley seed and may be equally useful for sterilising non-cereal species.

**Fig. 1:**
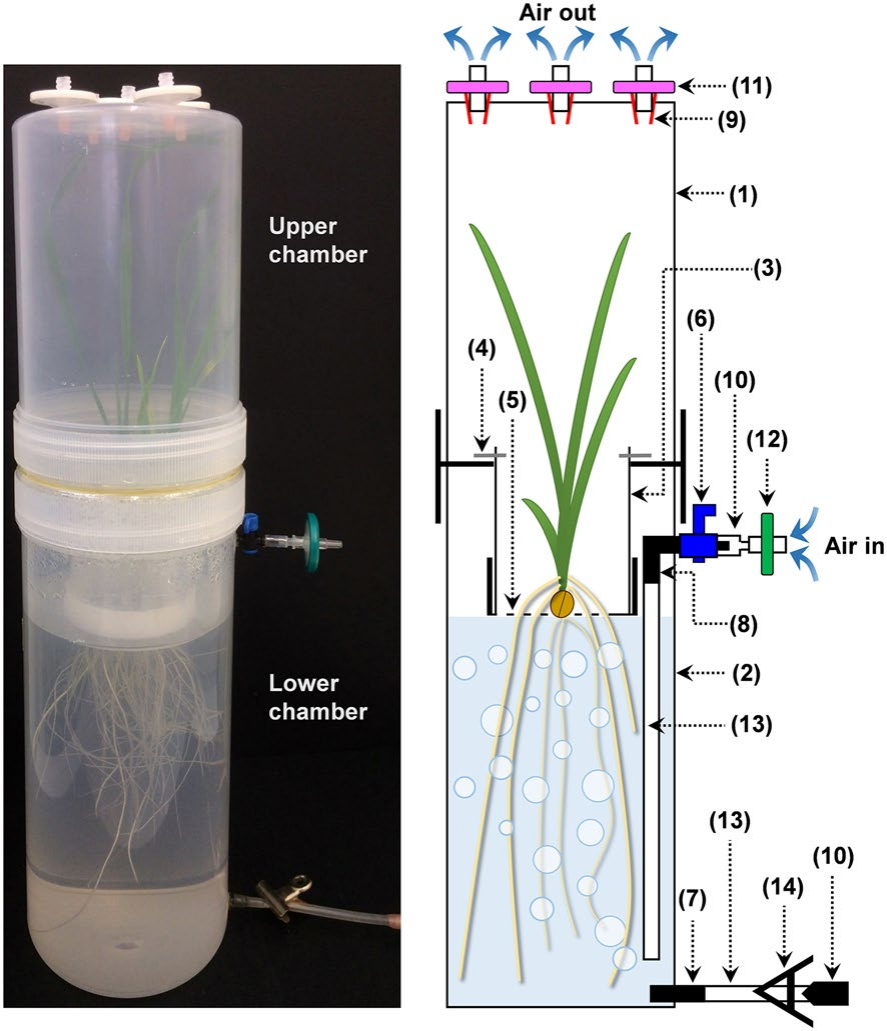
A photograph and a schematic drawing of the hydroponic system. The system is mainly comprised of an upper chamber that contains aerial part of the plant and a lower chamber that contains the nutrient solution and the plant root system. The plant is held between the two compartments with a plant holder insert. Numbers in bracket represent the part numbers listed in Table 2

Sterile-growth system: The system is fully enclosed and comprised of two joined chambers made from polypropylene containers or jars. The 2 L upper chamber covered the plant shoot and the 3 L lower chamber contained the roots and nutrient solution (Fig. 1). Detailed descriptions of the materials required and the procedure for constructing the apparatus is described in the Methods section. These chambers enabled the nutrient solutions to be aerated constantly and renewed regularly without microbial contamination. The polypropylene containers used to construct the system were slightly opaque and reduced the light intensity by ~ 18%. The light intensity of the growth cabinet was 610 μmol photon m^−2^ s^−1^ but the intensity inside the upper chamber was ~ 500 μmol photon m^−2^ s^−1^. However polypropylene containers were most suitable because, apart from being readily available and inexpensive, they withstand autoclaving, are easy to process (drill and glue) and release minimum amounts of organic substances.

Each hydroponic apparatus contained one pre-germinated wheat or barley seedling. The nutrient solutions were changed regularly and sterility was confirmed by periodically plating out a sample of nutrient solution on agar/nutrient plates. No bacterial contamination was detected in any of the samples but fungal contamination developed in two of the 30 systems assembled. The most likely source of this contamination was the seed. The composition of the nutrient solution is at the discretion of the experimenter. We used a simple low-strength nutrient solution (see “Methods” section) and nutritional status of the plants and pH of the nutrient solution were monitored. During the preliminary experiments solutions were renewed weekly and while no visual deficiency or toxicity symptoms were detected, tissue analysis at harvest indicated marginal P deficiency in some plants (Table 1). The solutions were changed more frequently in subsequent experiments. These changes also helped to maintain the solution pH which tended to decrease between solution changes (from pH 6.0 to 5.0). Changes in pH can also be minimised by adding a buffering agent or by modifying the composition of the nutrient solution (especially the ratio of ammonium and nitrate) depending on the experimental goals and the species being tested.

**Table 1:** Elemental analysis of hydroponically-grown wheat shoot tissue

Element	Hydroponics	Reference value^a^
Al (mg kg^−1^)	2.4 ± 0.5	< 200
B (mg kg^−1^)	4.4 ± 1.1	3–25
Ca (% w w^−1^)	0.20 ± 0.01	0.18–0.40
Cu (mg kg^−1^)	17.7 ± 0.5	1.3–18.0
Fe (mg kg^−1^)	63 ± 2	25–100
K (% w w^−1^)	3.2 ± 0.1	3.0–3.5
Mg (% w w^−1^)	0.08 ± 0.003	0.05–0.40
Mn (mg kg^−1^)	93 ± 6	< 700
P (% w w^−1^)	0.21 ± 0.02	0.24–0.70
S (% w w^−1^)	0.35 ± 0.01	0.28–0.30
Zn (mg kg^−1^)	102 ± 4	18–390

Whole-shoot tissues of sterile hydroponically grown 30-day-old wheat plants were homogenised and elemental content analysed by ICP-MS. Results show the mean ± SE (n = 10 plants)

^a^Reference values were based on the values (wheat at the Feekes scale 3 growth stage) previously reported [68]

**Fig. 2:**
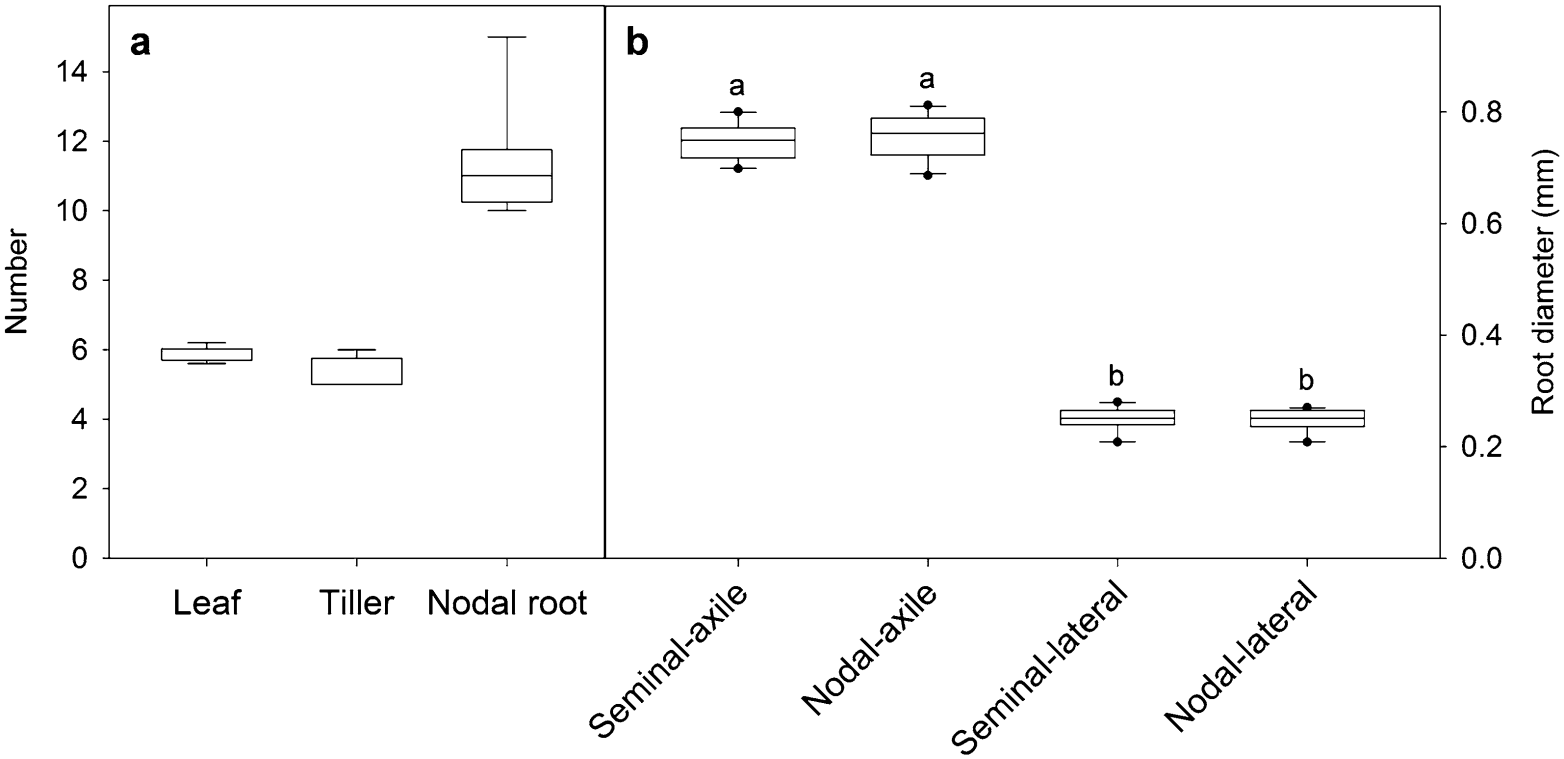
Wheat growth stage and root diameter in the hydroponic system. Box plots showing **a** the leaf number (leaf stage), number of tillers and the number of nodal roots developed on the hydroponic plants after 30 days of cultivation, and **b** the diameter of each root type of the hydroponically-grown wheat plants (n = 10). Different lowercase letters above the plots indicate significant differences as determined by a one-way ANOVA with Holm–Sidak post hoc test (*P* < 0.001)

**Fig. 3:**
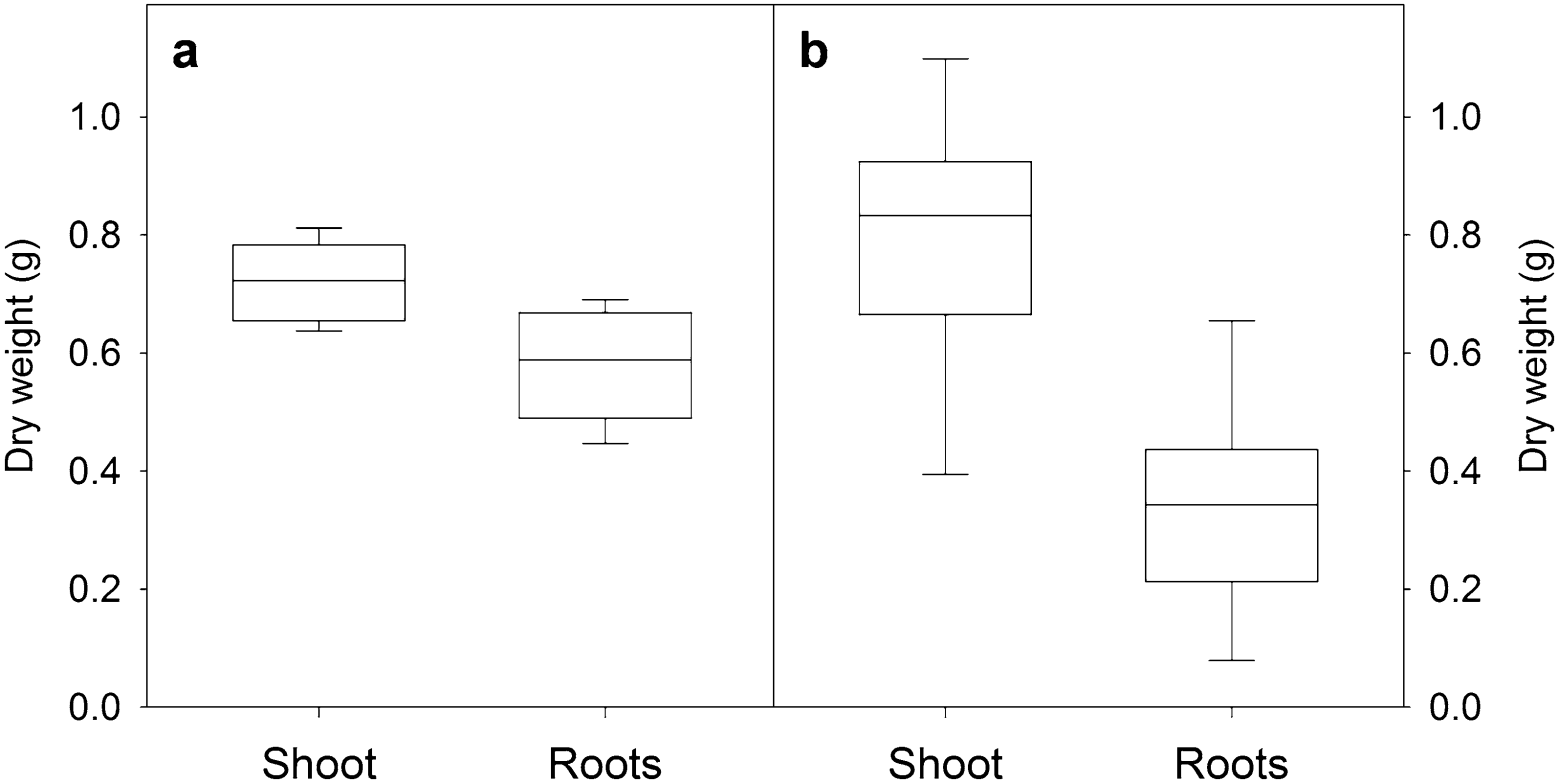
Biomass of hydroponically grown plants. Box plots showing the final shoot and root dry weight of **a** wheat (n = 10) and **b** barley (n = 8) after 30 days of cultivation in the sterile hydroponic system

*Plant growth* Wheat plants were typically grown until they were 30 days old. By this time they had reached the six-leaf stage with five to six tillers per plant (Fig. 2a). This is equivalent to the Zadoks scale of 25–26 (Feekes scale 3) [33]. The plants showed a relatively even growth between replicate growth systems with shoot dry weight ranging from 0.64 to 0.81 g per plant and root dry weight ranging from 0.45 to 0.69 g per plant (Fig. 3a). The two contaminated plants also developed biomass within the range. All wheat plants developed five seminal axile roots and between 10 and 15 nodal axile roots per plant (Fig. 2a). Diameters of the seminal and nodal axile roots were similar to one another and significantly greater than the laterals roots (*P* < 0.001) (Fig. 2b). Compared to the similar age plants grown in the non-sterile hydroponic system, the shoot biomass of the sterile plants was generally smaller (~ 10 to 20% less biomass) probably because they received slightly less light. Nevertheless the plants grown in both conditions developed similar root systems with comparable numbers of nodal and lateral roots (data not shown).

### Malate exudation from various root types of Al-resistant wheat

To demonstrate the utility of this sterile hydroponic system for analysing root exudates, we measured malate efflux from the various root types of mature (30 days old) wheat plants. The wheat cultivar EGA-Burke is resistant to Al^3+^ toxicity and shows the typical Al^3+^-activated efflux of malate from the seminal roots. This response has been characterised in detail in previous studies in a range of wheat cultivars [9, 24]. What was unknown, and the objective of these experiments, is whether the phenotype reported for seminal roots of young seedlings also occurs on the other root types on more mature plants.

**Fig. 4:**
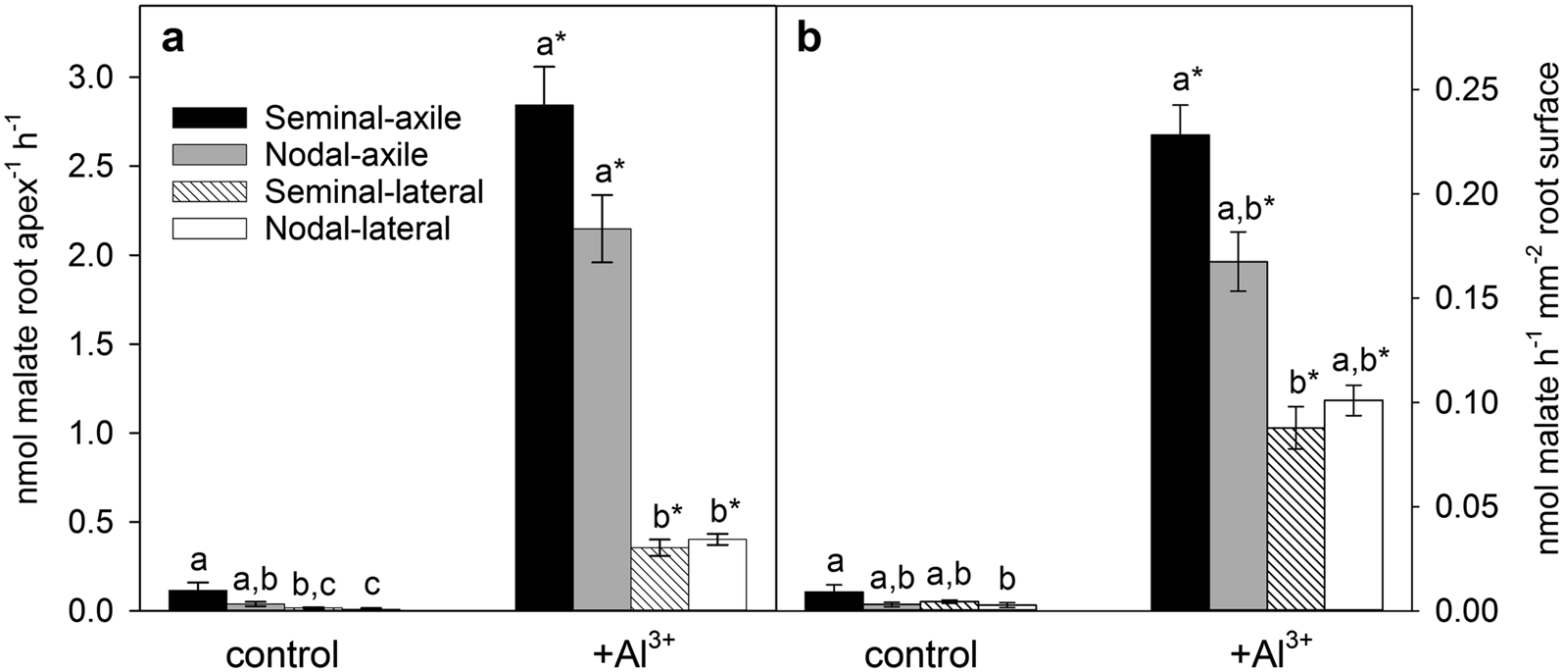
Malate efflux from the apices of various root types. Data are presented as **a** malate efflux per root apex, or **b** malate efflux normalized to the tissue surface area. Data are mean ± SE (n = 3–10). Lowercase letters above the bars indicate significant differences (*P* < 0.05) between the root types within the control or +Al^3+^ treatments, and asterisks above the +Al^3+^ bars indicate significantly more malate exudation (*P* < 0.05) than the counterpart root type in the control treatment. Equal variance tests failed so data were natural log-transformed prior to two-way ANOVA with Bonferroni post hoc test

After 30 days of growth, the plants were removed from the growth chambers, laid in clean trays with sterile nutrient solution so that the seminal, nodal and lateral roots could be identified. The root apices (5 mm) excised from these different root types were collected in a tube and used to measure malate efflux in the presence and absence of 60 μM AlCl_3_ (pH 4.3). In the control treatment (without Al^3+^), malate efflux was < 0.1 nmol apex^−1^ h^−1^ from all root types (Fig. 4a). In the presence of Al^3+^, malate efflux increased by 20 to 55-fold (*P* < 0.001) with the seminal and nodal axile roots releasing more than 2.0 nmol apex^−1^ h^−1^ and the lateral roots releasing ~ 0.40 nmol apex^−1^ h^−1^. Since root diameters of the axile roots (seminals and nodals) were three-fold greater than the lateral roots (Fig. 2b), the surface areas of these tissues will differ by a similar factor. When the fluxes are normalized for surface area, the malate efflux from axile and lateral roots was more similar and varied by two-fold only (Fig. 4b).

### Relative expression of *TaALMT1* in various root types

**Fig. 5:**
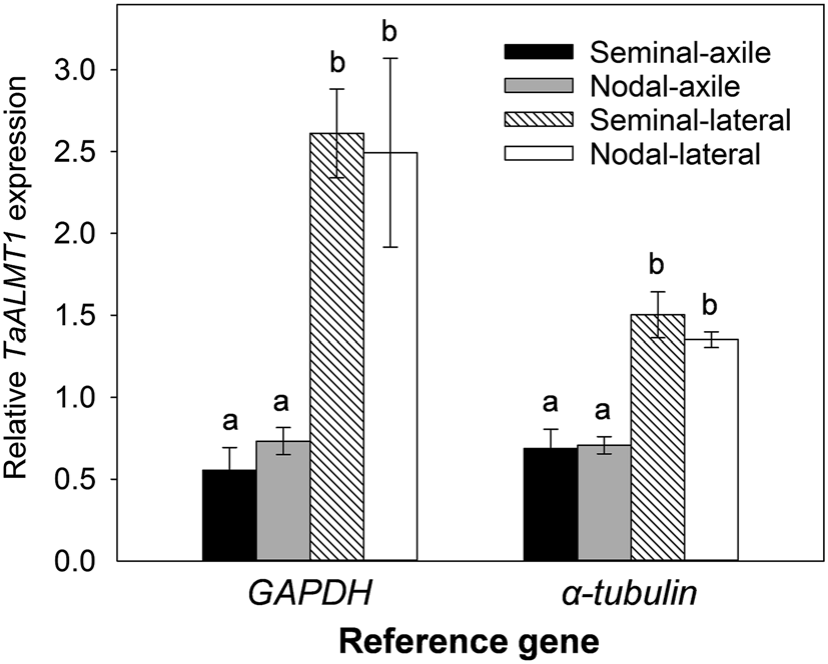
*TaALMT1* expression in various root types. Relative *TaALMT1* expression in the various roots of wheat were measured using *GAPDH* or *α*-*tubulin* as reference genes. Data from the control and +Al^3+^ treatments were combined. Data are mean ± SE (n = 6). Different lowercase letters indicate significant differences in the gene expression between the root types (*P* < 0.05) within each reference gene used. Equal variance test failed so data were natural log-transformed prior to one-way ANOVA with Holm–Sidak post hoc test

*TaALMT1* encodes an anion channel in wheat that facilitates the malate efflux measured above. Once the exudates were collected, RNA was extracted from tissues and *TaALMT1* expression was measured using quantitative RT-PCR (qRT-PCR) with two reference genes. *TaALMT1* expression was detected in all root types. Since *TaALMT1* expression was constitutive and unaffected by Al^3+^ treatment, data from the control and aluminium treatments were combined. Relative *TaALMT1* expression in the axile roots (seminal and nodal) was similar and expression in the lateral roots from the seminal and nodal roots was also similar (Fig. 5). However expression in the laterals was two to three-fold greater than in the axile roots using *α*-*tubulin* and glyceraldehyde 3-phosphate dehydrogenase (*GAPDH*) as reference genes, respectively.

### Organic exudates from whole root systems

To demonstrate another application of the hydroponic growth technique, we collected the exudates from the whole, intact root system of sterile barley plants. Barley (cv Golden Promise) plants grown in the hydroponic system for 30 days was slightly more vigorous than wheat and showed a greater variation in final root and shoot biomass (Fig. 3b). Roots were rinsed twice with sterile MilliQ water to wash off cell debris and nutrient solution, and root exudates were collected from the whole, intact root system in sterile MilliQ water for 2 h. Exudate compounds were identified with untargeted GC–MS analysis. The original nutrient solution cannot be analysed directly because the mineral salts are incompatible with GC–MS analysis. Furthermore, over time some exudates are likely to be reabsorbed by the roots. While not quantified in detail a total of 95 metabolites were detected among the barley root exudates with the major metabolite groups being amino acids (24 metabolites), organic acids (11 metabolites) or sugars and sugar derivatives (20 metabolites) (Additional file 1: Table S1). The experiment demonstrates another method for collecting exudates from sterile intact roots on more mature plants.

## Discussion

Root exudates are an important means by which plants interact with and control their environment. Hydroponic systems are widely used for studying root exudates even though they lack many of the chemical and mechanical stimuli that could affect growth and exudation [34, 35]. Notwithstanding the artificial nature of hydroponic systems, the literature contains many reports where the physiological processes described in hydroponically grown plants, and even sterile conditions, reflect the same processes occurring in soil-grown plants. Examples include the root exudates associated with aluminium resistance [9, 24, 25], nutrient stress [36, 37] and signalling. For instance, a diverse range of flavonoids released from roots in hydroponics are known to affect signalling, nodulation, plant development, pathogen defence and bacterial quorum sensing in the soil [38], and their roles as inducers of nodulation genes in rhizobia has been demonstrated in vitro using flavonoids collected from the exudates of hydroponically grown plants [39, 40]. Other links have been established between the exudates collected in hydroponics and the microbiome developed around soil-grown plants [41, 42].

Root exudates can be technically difficult to measure and most methods use seedlings or small plants (e.g. Arabidopsis) grown in hydroponic systems [43, 44]. Larger plants pose more challenges especially if sterile conditions are required to avoid microbial degradation of the exudates. Warren [31, 32] collected exudates from 4-week old wheat plants but since the shoots were not fully-enclosed it is unlikely that sterility of the roots was maintained. While the nutrient solutions were changed frequently in those experiments (every 2 to 6 days) the roots were maintained in a 250 mL container which is small considering the root biomass on wheat plants of that age.

The fully-enclosed hydroponic growth system described in this study could maintain sterility until wheat and barley plants were at least 30 days old by which time they had developed mature root systems. Five features of this method are emphasised: (1) Surface-sterilization of the seed is critical to avoid contamination; (2) The system maintained sterility in the chambers despite constant aeration and regular changes in nutrient solutions; (3) The large lower chamber allowed a relatively large volume (2.2 L) of nutrient solution to be included for root growth. This reduced the number of solution changes required which is important for minimising the risk of contamination; (4) The materials are inexpensive and the assembly is straight forward and uncomplicated; (5) The volume of the chambers can, in principle, be scaled up so that the plants can be grown for longer periods than described in this study.

Composition of the nutrient solution and pH can be adjusted to match the species or experiment requirements. It is important to maintain the pH stable because it influences the bioavailability of some nutrients [45]. A pH range of 5.5–6.5 is considered optimum for growing wheat in hydroponics [46]. We used 1 mM MES to buffer the nutrient solution to pH 6.0 but other buffers with different pKa values are available and the concentration of these can be increased to stabilise pH as long as growth is not adversely affected [47].

This growth system enabled us to characterise the Al^3+^-activated malate efflux from specific root types of mature wheat plants. This response is the major mechanism of Al^3+^ resistance in wheat [25] and although it has been studied in detail for 25 years, all previous reports have used young seedlings with seminal roots only. This has been an important omission because wheat seedlings only develop three to six seminal axile roots which represents a very small proportion of the final root length on mature plants. Nodal roots are probably 10 to 20-fold more numerous than seminal roots, while lateral roots represent approximately 95% of total root length in mature cereal plants [48]. Clearly these other roots types play a critical role in accessing water and nutrients [49, 50]. This sterile growth system enabled us to measure the malate efflux from the four major types of root (seminal axile, nodal axile, seminal lateral and nodal lateral) on 30-day-old wheat plants for the first time. We found that the Al^3+^-dependent malate release from seminal and nodal roots averaged 2.5 nmol root apex^−1^ h^−1^ which was greater than previous reports from seminal roots of 5-day-old seedlings [9, 24]. Lateral roots released less malate than the seminal and nodal axile roots, even after normalising for differences in root surface area (Fig. 4). These results demonstrate that the malate exudation from seminal roots is maintained for many weeks and that similar responses occur on the nodal and lateral roots. This study only investigated malate exudation but the system could be used to measure other exudates from various root types and in different genotypes.

*TaALMT1* is constitutively expressed in all four root types which is consistent with rapid activation of malate by Al^3+^ treatment from all the roots. Interestingly, expression level in lateral roots was significantly greater than the seminal or nodal axile roots even though malate efflux was less from the laterals. Future studies should compare TaALMT1 protein levels since protein/mRNA ratios for a given gene can be vary between tissues [51, 52].

The newly developed hydroponic system was also suitable for collecting exudates from whole-root systems. We demonstrated this by analysing the exudates from the whole root system of sterile 30-day-old barley plants. We collected root exudates by submerging the roots in 200 mL of MilliQ water in a 600 mL beaker with gentle shaking. This volume can be adjusted depending on the root biomass to minimize stress and damage to the tissue. Prolonged treatment to MilliQ water will damage roots because the integrity the root cell membranes can be compromised when Ca^2+^ is absent from the solution [53]. However the inclusion of too many salts in the solution poses other problems because once they become concentrated after lyophilisation they can interfere with the GC–MS analysis [54, 55]. As a compromise, we collected the exudates in MilliQ water but only for 2 h. This is shorter than many other studies, some of which collected exudates in ultra-pure water for up to 3 days [56]. Furthermore, the metabolite profile of root exudates collected in water or 0.5 mM CaCl_2_ was quantitatively and qualitatively similar after short collections periods [57].

Non-targeted GC–MS analysis identified a wide range of compounds with amino acids representing the largest metabolite group. This profile is generally consistent with other root exudate studies of barley and wheat grown in sterile sand [58], moistened filter paper [59] and in hydroponics [31, 32, 60, 61]. Even though exudates were collected over a relatively short period it is possible that the quantity of some compounds might be underestimated using this technique if the roots reabsorb them after being released [23, 31]. This system would also be useful for investigating specific root-microbe interactions in gnotobiotic conditions.

## Conclusions

We developed a novel hydroponic system that enabled cereal plants to be grown in sterile conditions for longer periods than previously recorded. Wheat and barley plants could be grown till they were 30 days old by which time they had reached the six-leaf stage and developed a mature root system. We demonstrated the advantages of the system for analysing exudates from wheat plants by measuring the Al^3+^-activated efflux of malate from seminal, nodal and lateral root apices for the first time. We also characterized the root exudates from whole, intact barley root systems. By providing a means of growing larger plants in sterile conditions for longer periods than previously described, this system overcomes the necessity for using smaller model species such as *Brachypodium* or *Arabidopsis* as surrogates for crop species.

## Methods

### Plant materials and media

The wheat (*Triticum aestivum* L.) used was the Al^3+^-resistant cultivar EGA-Burke which possesses the Type V allele of the *TaALMT1* gene associated with greater gene expression than Al^3+^-sensitive cultivars [62]. *TaALMT1* encodes the Al^3+^-activated efflux of malate from the root apices. The barley (*Hordeum vulgare* L.) cultivar used was Golden Promise.

The hydroponic nutrient solution contained 0.5 mM CaCl_2_, 150 μM MgSO_4_, 1 mM KNO_3_, 0.5 mM NH_4_Cl, 2 μM Fe EDTA, 10 μM KH_2_PO_4_, 11 μM H_3_BO_3_, 2 μM MnCl_2_, 0.35 μM ZnSO_4_, 0.05 μM (NH_4_)_6_Mo_7_O_24_ and 0.2 μM CuCl_2_. The pH of the nutrient solution was adjusted to 6.0, and buffered with 1 mM MES. Microbial contamination of seed and nutrient solution was checked by placing them on plates containing 1 × Nutrient Broth (Difco) solidified with 1.5% agar.

### Seed sterilization

The following procedure for surface-sterilizing the seed was mostly performed in a laminar flow hood. The seeds were first washed with 70% EtOH for 1 min and then imbibed for 7 h at room temperature (RT) in a 50 mL screw-top tube half-filled with sterile water plus 0.05% Tween 20. The tube was place on a shaker during this period (~ 50 rpm). Barley seeds were dehusked prior to this stage. The solution was poured out and the imbibed seed were treated in 20% household bleach for 10 min on a shaker followed by multiple rinses with sterile water. The solution was then replaced with 3% H_2_O_2_ and placed on a shaker for 30 min [63]. After thorough rinses in sterile water, the tube was half filled with sterile water heated to 50 °C, sealed and placed in a 50 °C water bath for 10 min [64]. Sterilized seeds were then placed on Nutrient Broth agar plates, stratified at 4 °C for 24 h and allowed to germinate for 2 days at room temperature. Only seedlings with no visible contamination were transferred to the hydroponic system. An additional treatment with systemic fungicide or bactericide could be included if the surface sterilization of seed is insufficient to prevent contamination occurring.

### Hydroponic system construct

The materials required to construct one hydroponic system are listed on Table 2 and shown in Additional file 1: Figs. S2 and S3. The hydroponic system used two containers to create the two main chambers (Fig. 1). The 2 L upper chamber enclosed the aerial part of the plant (Part #1, Table 2). The 3 L lower chamber contains the nutrient solution and plant root system (Part #2).

**Table 2:** Materials for constructing one hydroponic system

Part #	Material	Quantity	Supplier
1.	2 L polypropylene clear plastic container with a screw lid (13 cm diameter × 19 cm tall) (product code: JARC2000)	1	The Plastic Man, Mordialloc, VIC, Australia
2.	3 L polypropylene clear plastic container with a screw lid (13 cm diameter × 28.5 cm tall) (product code: JARC3000)	1	
3.	500 mL polypropylene clear tube with screw cap (6.7 cm diameter × 15 cm tall) (product code: 75.9922.812)	1	Sarstedt
4.	Stainless steel wire (1.6 mm diameter), cut into 1 cm length	6	N/A
5.	Polyethylene plastic mesh (3 mm), cut into 6.5 cm diameter disc	1	Menzel Plastic Traders, Melrose Park, SA, Australia
6.	4 mm Threaded inline tap (product code: 1010452)	1	Pope Products, Beverley, SA, Australia
7.	4 mm Threaded adaptor (product code: 1010013)	1	
8.	4 mm Elbow adaptor. One side was trimmed off and the hole was enlarged, so it can be connected to the Part #6 (product code: 1010007)	1	
9.	Syringe needle. The needle was removed, and only the plastic attachment base was used	5	Terumo
10.	1 mL Syringe. Only the first 2 cm of the syringe head was used. The rest was cut off and removed. This can also be used as a plastic plug when the hole sealed is sealed	2	
11.	0.45 μm Nylon syringe filter (autoclavable) (product code: ESF-NY-30-045)	5	Kinesis Australia, Redland Bay, QLD, Australia
12.	0.22 μm PES sterile syringe filter (product code: Z359904)	1	Millipore
13.	Medical grade silicone tubing (4 mm diameter), cut into 10 cm and 20 cm length	1 Each	N/A
14.	Metal bulldog clip	1	N/A
15.	Air compressor (35 L min^−1^ capacity), (product code: HAILEA ACO-208)	1	Guandong Hailea Group, Guandong, China
16.	Aquarium pump tubing (4 mm diameter) and plastic T-joints for the tubing	As required	N/A
17.	Plastic glue (J-B Weld Plastic Bonder Syringe)	As required	J-B Weld, Sulphur Springs, TX

Product codes were supplied whenever possible. Parts can be replaced with other suitable materials

- The lids to these containers (Parts #1 and #2) were smoothed with a rotary tool (Dremel), then their upper surfaces scraped to make them rough so the two lids could be glued together with a plastic glue (Part #17) (Additional file 1: Fig. S4a). This glue is able to bond polypropylene and is resistant to autoclaving. Once the glue was dry, a 7 cm diameter hole was bored in the centre of the glued lids (Additional file 1: Fig. S4b) which is where the plant holder will sit.
- The plant holder was made from a 500 mL polypropylene container with lid (Part #3). The bottom 7 cm of the tube was removed using a saw, and four small pieces of stainless steel wire (Part #4) were heated and inserted through the plastic around the cut edge (Additional file 1: Fig. S5a), so that the plant holder could later be suspended upside down in the hole made in the glued lids (Additional file 1: Fig. S6). A hole (6 cm diameter) was bored in the centre of the screw cap of the polypropylene tube (Part #3). This allowed a disc of plastic mesh (Part #5) to sit on the inside when the tube was suspended from the glued lids. To secure the mesh in place, two additional pieces of stainless steel wire (Part #4) were heated and inserted through the side of the lid (Additional file 1: Fig. S5b).
- Five 6 mm diameter holes were drilled in the bottom of the upper chamber (Part #1). These later held filters to vent air from the upper chamber. Firstly, syringe needles were obtained and the metal needles removed from their plastic bases by sawing or cutting. These bases (Part #9) were inserted into the drilled holes and glued in place so that 0.45 μm nylon syringe filters (Part #11) could be later inserted on the outside (Additional file 1: Fig. S7).
- Two 4 mm holes were drilled on the side of the lower chamber (Part #2). The first hole was drilled 4 cm from the top for the air inflow to aerate the solution. The second hole was drilled near the bottom of the chamber and was used for draining when the nutrient solution required changing (see Fig. 1). An inline plastic tap (Part #6) was screwed into the upper air inlet hole and glued in place. Once dry an elbow adaptor (Part #8) was firmly attached and glued to the tap on the inner side of the chamber. On the outer side of the chamber, a syringe head (Part #10) was attached and glued to the inline tap (Part #6), and this allowed a syringe filter (Part #12) to be attached for filtering the incoming air. A 20 cm length of silicon tubing (Part #13) was attached to the elbow adapter (Part #8) so that the lower end of the tubing was near the bottom of the chamber. A 4 mm threaded adaptor (Part #7) was screwed into the bottom draining hole and glued in place. A ~ 10 cm length of silicon tubing (Part #13) was attached to the draining adaptor (Part #7) at the bottom of the jar. This draining tube was plugged with a plastic plug, which can be made from a 1 mL syringe head that had the hole was sealed with heat (Part #10), and clamped with a metal bulldog clip (Part #14).
- The whole system can be pre-assembled, filled with nutrient solution, and then autoclaved, or each component can be wrapped in foils, autoclaved separately and then assembled in the laminar flow hood.

### Hydroponic system setup and plant growth condition

All sterile setups were performed in a laminar flow cabinet. A 0.22 μm sterile nylon syringe filter (Part #12) was attached to the air inlet tap (Part #6 and 10 joint) to filter the inward-flowing air (Fig. 1). A series of 0.45 μm sterile nylon syringe filters (Part #11) were then inserted into the adaptors (Part #9) at the top of the upper chamber (Additional file 1: Fig. S7).

Sterile nutrient solution (~ 2.2 L) was added to the lower chamber until the solution just touched the bottom of the plant holder when it was inserted and suspended from the hole in the glued lids (Additional file 1: Fig. S6). The lower chamber was screwed into the glued lids, and using sterile forceps, a surface-sterilized, germinated seedling was carefully placed on the middle of the plastic mesh of the plant holder so that its young roots passed through the mesh and into the nutrient solution. The upper compartment was then screwed into the other side of the glued lids. The compartments and the glued lids were sealed with Parafilm.

Plants were cultivated in a growth cabinet (Conviron, Canada) with a 16 h day/8 h night cycle (20 °C/15 °C) and 610 μmol photon m^−2^ s^−1^ light intensity. The light intensity was attenuated in the polypropylene containers by ~ 18%. The lower chamber was not covered so that root development could be easily monitored. However, if it is important that the nutrient solution not be exposed to light the lower chamber can easily be covered with foil or cloth. Typically 12 hydroponic setups were prepared at one time. Aquarium pump tubing (Part #16) of equal length were attached from the 0.22 µm filters to a manifold which was connected to an air compressor (Part #15). The tube connecting the manifold to the air compressor first passed air through a closed chamber containing activated carbon granules which removed organic volatiles emitted from the pump. The equal length of tubing between the manifold and each inlet port helped ensure the air pressure going to each apparatus was equal. Final aeration rates were 2.9 L air min^−1^.

Nutrient solutions required changing once or twice a week depending on the size of the plants but this will vary depending on the species and the composition of the nutrient media. To change the solutions the aeration tubes were detached from the filters and each apparatus transferred to a laminar flow hood. The solutions were drained into a container via the bottom tube by removing the bulldog clip and the plug (this occurred more easily if the lower chamber is unscrewed slightly). The plug was sterilized with 70% EtOH each time it was removed from the tube. Once the solution was drained, the draining tube was plugged and clipped again and the lower chamber unscrewed from the lid. Fresh sterile nutrient solution was added to the lower chamber, by lifting the upper chamber (with the plant holder and plant) for a short time. At each change the pH of the used solution was measured and sterility checked by plating out a sample on Nutrient Broth agar plates and incubating them at 25 °C for 3 days.

### Plant sampling and root exudate collection

Wheat and barley plants were harvested after another 28 days of cultivation in the hydroponic system (30 days old from germination). To measure malate exudation from various wheat roots, the upper and the lower chambers were detached, and plants carefully removed from the plant holder and laid on a tray with sterile nutrient solution. The mesh disc remained attached to the roots. The plant was scored for growth stage, tiller number and nodal root number. All axile roots (nodal and seminal) were cut off from the base of the stem and washed in sterile 0.2 mM CaCl_2_ solution. The root apices (5 mm) of seminal axile roots, nodal axile roots and the primary lateral roots of seminal and nodal roots were excised with a scalpel and placed in a glass tube. Malate released from these root apices was collected as described previously [24]. Each replicate included five root apices from seminal axile roots, 10-15 root apices from nodal axile roots, and 30 root apices from each lateral root type. They were washed with 1 mL of 0.2 mM CaCl_2_ (pH 4.3) in 5 mL glass vials sealed with Parafilm by placing the vials on their sides on a platform shaker (~ 50 rpm) for 30 min. The washing solution was removed, and the root apices were rinsed twice with 1 mL of 0.2 mM CaCl_2_ (pH 4.3) (control treatment) or with 0.2 mM CaCl_2_ with 60 μM AlCl_3_ (pH 4.3) (+Al^3+^ treatment). Finally, 1 mL of 0.2 mM CaCl_2_ (pH 4.3) or 60 μM AlCl_3_ (in 0.2 mM CaCl_2_, pH 4.3) was added to the vials which were then sealed and returned to the shaker for 2 h. The root exudate solutions were removed and stored at − 20 °C until the subsequent organic anion analysis. The remaining root apex tissues were stored at − 80 °C for later RNA extraction. The diameter of each root type was later measured from stereo microscope images.

To collect exudates from the whole root system of sterile 30-day-old barley plants, the lower chamber of the apparatus was detached and the root system rinsed twice in sterile MilliQ water to wash off the cell debris and nutrient solution. The root system was placed in a 600 mL glass beaker with 200 mL of sterile MilliQ water so that the roots were fully immersed (Additional file 1: Fig. S1). The whole apparatus was placed on a gentle shaker and exudates collected for 2 h. The plants were removed and the solutions filtered with a 0.22 μm PHENEX RC syringe filter (Phenomenex) and stored at − 20 °C for subsequent analyses by gas chromatography-mass spectrometry (GC–MS).

The shoots and remaining roots were dried in the oven (65 °C) and the dry weights were measured. If elemental analysis of shoots was required, the dried material was acid digested with microwave heating with a Milestone-Start D Microwave Digestion System (Milestone Inc., CT, USA) according to the US EPA method 3015 [65], and the elemental analysis performed with inductively coupled plasma mass spectrometry (ICP-MS) using a Varian Vista-Pro inductively coupled plasma-optical emission spectrometer (Varian Inc., CA, USA) (Analytical Chemistry Group, CSIRO, Australia).

### Measurement of malate exudation from root apices

Malate efflux from the root apices was quantified enzymatically. The procedure was similar to previous descriptions [25] except that NADH was measured by fluorescence as follows. An aliquot of the exudate solution (5–10 μL) was mixed with 50 μL of hydrazine buffer (0.5 M glycine, 0.4 M hydrazine, pH 9.0), 5 μL of 40 mM NAD and placed in a black 96-well plate. Water was added to a final volume of 105 μL. The initial relative fluorescence unit (RFU) was obtained using a TECAN Infinite M200 PRO microplate reader with 340 nm excitation wavelength and 440 nm emission wavelength. Malic dehydrogenase (0.3 μL, ~ 1.4 units) (Sigma-Aldrich) was added to each sample and mixed with a pipette and incubated for 30 min at RT. The RFU readings were measured again using the same settings. The increase in the RFU due to production of NADH is proportional to the initial amount of malate in the sample. A standard curve generated with a range of malate concentrations was used to estimate malate concentrations in the samples.

### GC–MS analysis of root exudates from barley whole root system

Aliquots (50 mL) of the barley root exudates were lyophilized and resuspended in 2 mL of methanol:H_2_O = 1:1 (vol:vol), centrifuged at 16,100 g for 5 min to remove solid matter, and 400 μL of the resulting supernatant was dried down in 2 mL amber glass vials with inserts using a MiVac centrifugal concentrator (SP Scientific). Dried samples were methoxymated with 15 μL of 20 mg mL^−1^ methoxyamine hydrochloride (Sigma-Aldrich) in pyridine at 37 °C for 90 min, followed by silylation with 15 μL of *N*-methyl-*N*-(trimethylsilyl)-trifluoroacetamide (GC derivatization grade, Sigma-Aldrich) at 37 °C for 30 min. Derivatised samples were analysed by GC–MS on a DB5 column (0.25 mm internal diameter, 1 μm film thickness, 30 m length) with a Shimadzu TQ8050 GC–MS/MS (Shimadzu Corp.) in multiple reaction monitoring (MRM) mode. Sample (1 μL) was injected in split mode at a 1:10 ratio into the inlet that was heated to 280 °C, and helium was used as the carrier gas at a constant linear velocity of 39 cm s^−1^ with a purge flow rate of 5 mL min^−1^. The GC oven was held at 100 °C for 4 min, followed by a 10 °C min^−1^ ramp to 320 °C, which was held for 11 min. The MS interface and ion source temperatures were held at 280 °C and 200 °C, respectively, and the ionisation voltage was 70 eV. Metabolites were detected using the Smart Metabolites Database (Shimadzu Corp.), which contains MRM parameters for optimal transitions and retention time window for 467 metabolites. Detected metabolites in MRM mode were cross-checked by analysing the derivatised samples in full scan mode with the mass measurement range between 45 and 600 m/z, and mass spectrum of peaks of interest searched against NIST 14 (National Institute of Standards and Technology, U.S. Department of Commerce) and the Golm metabolome database (http://gmd.mpimp-golm.mpg.de/).

### RNA extraction and quantitative Real-Time PCR

RNA was extracted from the wheat root apices using the RNeasy Plant Mini Kit (QIAGEN) according to the manufacturer’s protocol, which included a DNase step. cDNA was synthesized using SuperScript IV Reverse Transcriptase (Thermo Fisher Scientific) with Oligo(dT)_20_ primer (Thermo Fisher Scientific). Gene expression of *TaALMT1* (malate anion channel) was analysed with qRT-PCR. Primers CGTGAAAGCAGCGGAAAGCC (forward) and CCCTCGACTCACGGTACTAACAACG (reverse) [66] were used to amplify the *TaALMT1* transcript. *GAPDH* and *α*-*tubulin* were used as the reference genes, and these were amplified with TCAGACTCCTCCTTGATAGC (forward) and GTTGAGGGTTTGATGACCAC (reverse) primer set [67], and TCCAGTTCGTCGACTGGTGC (forward) and TCCTCGTAGTCCTTCTCCAG (reverse) primer set [8] respectively. qRT-PCR was performed with a Lightcycler^®^ 480 Instrument II (Roche), in a 10 μL reaction mix consisted of 5 μL SsoAdvanced™ Universal SYBR^®^ Green Supermix (Bio-Rad), 5 pmol each of forward and reverse primers and 10 ng of the template cDNA. The PCR conditions were 95 °C for 5 min, followed by 45 cycles of 95 °C for 15 s, 55 °C for 20 s and 72 °C for 30 s (+ plate read). Melt-curve analysis was performed after the amplification step by increasing the temperature from 65 to 95 °C (0.5 °C increment, 5 s per step) (+ plate read).

### Statistical analysis

One-way analysis of variance (ANOVA) and two-way ANOVA were performed with SigmaPlot 13.0 package (Systat Software Inc., San Jose, CA). When the data showed non-homogeneous variances using the Brown-Forsythe test, the data were transformed using the natural logarithms prior to the statistical analysis. Post hoc analysis were performed with either Bonferroni or Holm-Sidak methods depending on the data set.

## Additional file

**Additional file 1: Table S1**. Identity of exudates collected from the whole root system of 30-day-old sterile-grown barley plants.** Figure S1**. Method for collecting exudates from the whole root system of sterile-grown barley plants.** Figure S2**. A photograph of some components used for constructing the sterile hydroponic system.** Figure S3**. Photographs of other components used for constructing the sterile hydroponic system.** Figure S4**. Connector of the upper and the lower chambers of the hydroponic system.** Figure S5**. Details of the plant holder.** Figure S6**. A plant holder suspended from the glued lids.** Figure S7**. View from above of the upper chamber and the air vents of the hydroponic system.
