# Supplementary material for: A sterile hydroponic system for characterising root exudates from specific root types and whole-root systems of large crop plants
Source: Plant Methods. 2018 Dec 20;14:114. doi: 10.1186/s13007-018-0380-x (PMC6300921; doi:10.1186/s13007-018-0380-x)
Supplement: Supplementary file 1 — Additional file 1: Table S1. Identity of exudates collected from the whole root system of 30-day-old sterile-grown barley plants. Figure S1. Method for collecting exudates from the whole root system of sterile-grown barley plants. Figure S2. A photograph of some components used for constructing the sterile hydroponic system. Figure S3. Photographs of other components used for constructing the sterile hydroponic system. Figure S4. Connector of the upper and the lower chambers of the hydroponic system. Figure S5. Details of the plant holder. Figure S6. A plant holder suspended from the glued lids. Figure S7. View from above of the upper chamber and the air vents of the hydroponic system. [file 13007_2018_380_MOESM1_ESM.docx]

**SUPPLEMENTARY MATERIALS**

**Kawasaki et al**

**Table S1. Identity of exudates collected from the whole root system of 30-day-old sterile-grown barley plants.**

| Class | Metabolite |
| --- | --- |
| Amino acid | 2-Aminopimelic acid |
|  | 3-Aminoglutaric acid |
|  | 3-Aminopropanoic acid |
|  | 3-Sulfinoalanine |
|  | 4-Aminobutyric acid |
|  | 5-Oxoproline |
|  | Alanine |
|  | Asparagine |
|  | Aspartic acid |
|  | Cystine |
|  | Glutamic acid |
|  | Glutamine |
|  | Glycine |
|  | Isoleucine |
|  | Leucine |
|  | Lysine |
|  | N-Acetylserine |
|  | Phenylalanine |
|  | Proline |
|  | Serine |
|  | Threonine |
|  | Tryptophan |
|  | Tyrosine |
|  | Valine |
|  |  |
| Organic acid | Aconitic acid |
|  | Citramalic acid |
|  | Citric acid |
|  | Fumaric acid |
|  | Glycolic acid |
|  | Isocitric acid |
|  | Lactic acid |
|  | Maleic acid |
|  | Malic acid |
|  | Oxalic acid |
|  | Succinic acid |
|  |  |
| Sugar and sugar derivative | 1,6-Anhydroglucose |
|  | 2-Deoxy-glucose |
|  | Arabinose |
|  | Fructose |
|  | Ribonolactone |
|  | Sorbose |
|  | Sucrose |
|  | Trehalose |
|  | Glyceric acid |
|  | Threonic acid |
|  | Arabitol |
|  | Glycerol |
|  | Inositol |
|  | Mannitol |
|  | meso-Erythritol |
|  | Ribitol |
|  | Sorbitol |
|  | Threitol |
|  | Xylitol |
|  | Dihydroxyacetone phosphate |
|  |  |
| Fatty acid | Caproic acid |
|  | Elaidic acid |
|  | Myristic acid |
|  | Palmitic acid |
|  | Stearic acid |
|  |  |
| Carboxylic and dicarboxylic acid | Nicotinic acid |
|  | Phenylacetic acid |
|  | 2-Ketoglutaric acid |
|  | Azelaic acid |
|  | Glutaric acid |
|  | Pimelic acid |
|  |  |
| Nucleoside and nucleotide | Adenosine |
|  | Guanosine |
|  | Adenine |
|  | Cytosine |
|  | Guanine |
|  | Thymine |
|  | Uracil |
|  |  |
| Hydroxy and hydroxycarboxylic acid | 2-Hydroxyglutaric acid |
|  | 3-Hydroxyglutaric acid |
|  | Pantothenic acid |
|  | 2-Hydroxybutyric acid |
|  | 3-Hydroxyisobutyric acid |
|  | 3-Hydroxyisovaleric acid |
|  | 3-Hydroxypropionic acid |
|  |  |
| Others | Glycerol 3-phosphate |
|  | Xanthine |
|  | Octopamine |
|  | 2-Aminoethanol |
|  | 3-Methoxy-4-hydroxybenzoic acid |
|  | 4-Hydroxybenzoic acid |
|  | Glucaric acid |
|  | Gluconic acid |
|  | Galacturonic acid |
|  | Phosphoric acid |
|  | Ribonic acid |
|  | Hydroquinone |
|  | Putrescine |
|  | Glucono |
|  | Dihydrouracil |
|  |  |

Only metabolites that were detected in at least 2 out of 8 biological replicates listed here.


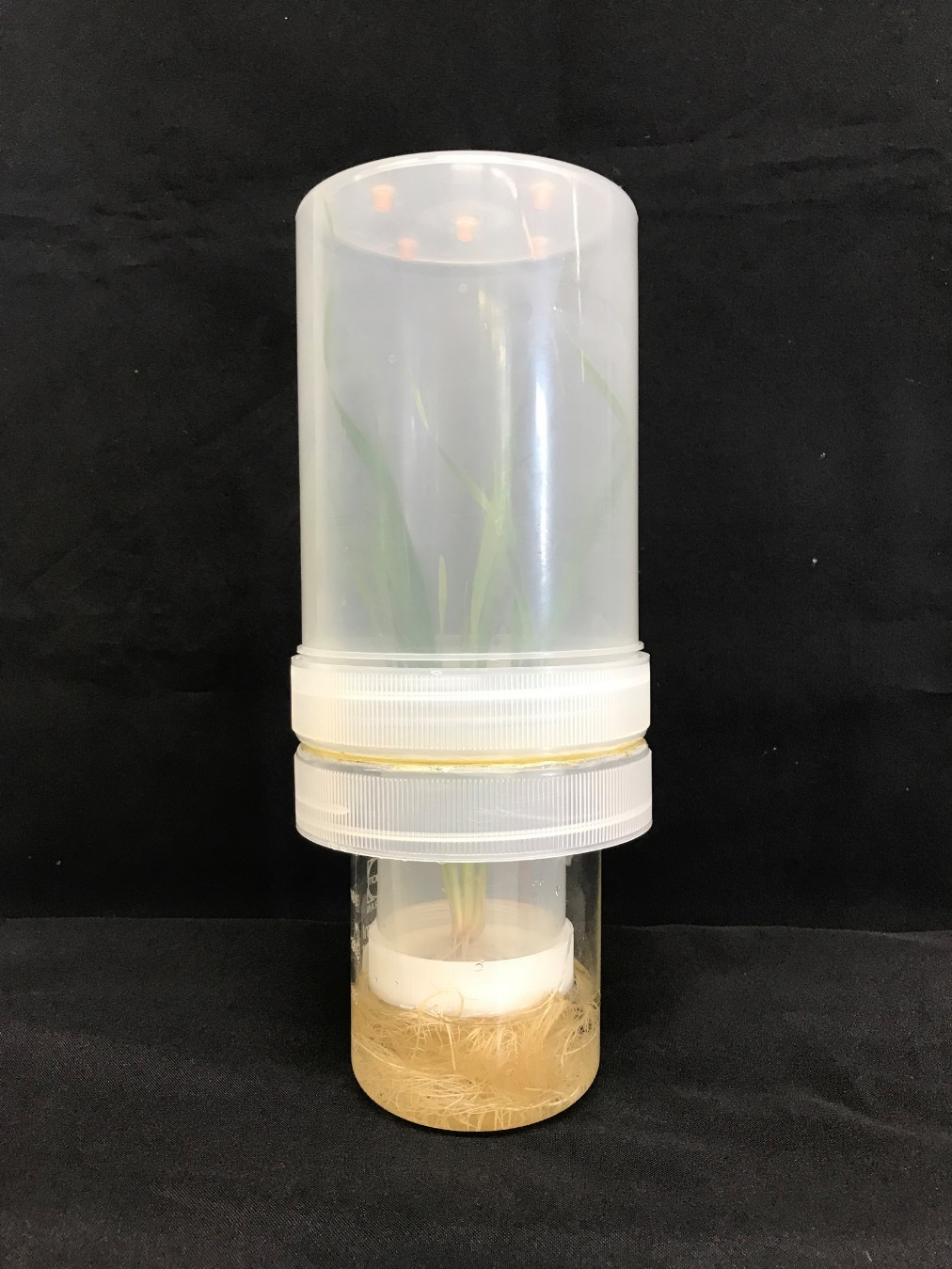


**Fig. S1. Method for collecting exudates from the whole root system of sterile-grown barley plants.**

The whole root system were rinsed twice with sterile MilliQ water to remove cell debris and nutrient solution, and then immersed into 200 mL of sterile MilliQ water in a 600 mL beaker. The chambers were placed on a gentle shaker and exudates were collected over 2 h period.

**Fig. S2. A photograph of some components used for constructing the sterile hydroponic system.**

Numbers in bracket represent the part numbers listed in Table 2.

**Fig. S3. Photographs of other components used for constructing the sterile hydroponic system.**

Numbers in bracket represent the part numbers listed in Table 2.

**Fig. S4. Connector of the upper and the lower chambers of the hydroponic system.**

Two lids of the polypropylene jars (Parts #1 and #2) were glued, and a hole was bored in the centre of the glued lids so that the plant holder (Part #3) can be inserted. Views from the side (a) and the top (b). Numbers in bracket represent the part numbers listed in Table 2.

**Fig. S5. Details of the plant holder.**

(a) The bottom 7 cm of the 500 ml polypropylene tubes (Part #3) was removed and four small pieces of stainless steel wires (Part #4) were heated and inserted into the side around the cut edge. (b) A hole (6 cm diameter) was made in the centre of the screw cap, and a plastic mesh disc (Part #5) was placed on the inside. Two pieces of stainless steel wire (Part #4) were heated and inserted in the side of the screw cap to prevent the plastic mesh from moving. Numbers in bracket represent the part numbers listed in Table 2.

**Fig. S6. A plant holder suspended from the glued lids.**

(a) View of the plant holder from the side (lower chamber removed), and (b) from above (upper chamber removed). Numbers in bracket represent the part numbers listed in Table 2. The wire pins (Part #4) allow the plant holder to suspend from the 7 cm diameter hole on the glued lids.

**Fig. S7. View from above of the upper chamber and the air vents of the hydroponic system.**

Five holes were drilled on the bottom of 2 L jar (Part #1), and plastic attachment bases of syringe needles (Part #9) were glued in to the holes. Filters (Part #11) were attached in these holes. Numbers in bracket represent the part numbers listed in Table 2.
